# Stepped care for depression is easy to recommend, but harder to implement: results of an explorative study within primary care in the Netherlands

**DOI:** 10.1186/1471-2296-15-5

**Published:** 2014-01-09

**Authors:** Marleen LM Hermens, Anna Muntingh, Gerdien Franx, Peter T van Splunteren, Jasper Nuyen

**Affiliations:** 1 Netherlands Institute for Mental Health and Addiction (Trimbos Institute), PO Box 725, 3500 AS Utrecht, the Netherlands; 2 GGZ Ingeest, PO Box 74077, 1070 BB Amsterdam, the Netherlands

**Keywords:** Qualitative research, Primary health care, General practice, Depression, Stepped care, Collaborative care

## Abstract

**Background:**

Depression is a common mental disorder with a high burden of disease which is mainly treated in primary care. It is unclear to what extent stepped care principles are applied in routine primary care. The first aim of this explorative study was to examine the gap between routine primary depression care and optimal care, as formulated in the depression guidelines. The second aim was to explore the facilitators and barriers that affect the provision of optimal care.

**Methods:**

Optimal care was operationalised by indicators covering the entire continuum of depression care: from prevention to chronic depression. Routine care was investigated by interviewing general practitioners (GPs) individually and together with other mental health care providers about the depression care they delivered collaboratively. Qualitative analysis of transcripts was performed using thematic coding. Additionally, the GPs completed a self-report questionnaire.

**Results:**

Six GPs and 22 other (mostly primary) mental health care providers participated. The GPs and their primary care colleagues embraced a general stepped care approach. They offered psycho-education and counselling to mildly depressed patients. When the treatment effects were not satisfactory or patients were more severely depressed, the GPs offered, or referred to, psychotherapy or pharmacotherapy. Patients with a complex and severe depressive disorder were directly referred to specialised mental health care. However, GPs relied on their clinical judgment and rarely used instruments to assess and monitor the severity of depressive symptoms. Structured, evidence based interventions such as self-management and e-health were rarely offered to patients with depressive symptoms. Specific psychological interventions for relapse prevention or for chronically depressed patients were not available. A wide range of influencing factors for the provision of optimal depression care were put forward. Close collaboration with other mental health care professionals was considered an important factor for improvement by nearly all GPs.

**Conclusions:**

The management of depression in primary care seems in line with stepped care principles, although it can be improved by applying more elements of a stepped care approach. Collaboration between GPs and mental health care providers in primary care and secondary care should be enhanced.

## Background

Depression is a common mental disorder. Approximately 13% of the population in European countries experience depression at least once in their life [1]. Depression has a high burden of disease [2,3] and a high economic burden [4]. In the Netherlands, as in other European countries, the majority of people suffering from depression receive treatment in primary care [5,6].

The Netherlands has a strongly developed primary care system. There are approximately 11,000 GPs in the Netherlands [7]. Nearly all Dutch citizens (98%) are registered with a general practice where they receive care that is free of charge, and most patients have long lasting contact (>10 years) with the same general practitioner (GP). Besides GPs, other professionals provide primary mental health care. These include physiotherapists, nurses, social workers and psychologists. The GP is the central provider for all primary care, including mental health care, and acts as a gatekeeper to secondary (specialised) care.

Worldwide, integrating mental health services into primary care is increasingly considered the most viable way to ensure access to mental health care [8]. New cost containment policies in the Netherlands have recently urged GPs to adopt an even larger role in treating mental health conditions and to refrain from referring patients to specialised mental health services. The deployment of a mental health nurse (MHN) in primary care in 2008 was one of the most important recent measures to strengthen primary mental health care. Most MHNs are trained as psychiatric nurses and they assist GPs in the care for patients with mental health problems. In 2011, about one third of GPs in the Netherlands collaborated with an MHN [9].

Many patients with a non-severe or non-complex depression receive mental health care in primary care [5,6]. Several guidelines are available for Dutch GPs to deliver evidence based depression care. An over-arching multidisciplinary clinical guideline on depressive disorders [10] is available for all professionals providing mental health care in primary care or in specialist care, including GPs. This multidisciplinary guideline recommends a stepped care approach based on the severity and duration of the depressive episode. Recently, a revision of the monodisciplinary guideline on depression, developed by the Dutch College of General Practitioners (NHG), was published [11]. The revised NHG-guideline corresponds largely with the multidisciplinary guideline and recommends the same stepped care principles.

Internationally, stepped care models have been adopted in several recently developed clinical guidelines on depression [12,13]. The stepped care model offers a range of several effective treatments. Depending on the severity of the depression, a treatment is allocated, starting with the least intensive treatment that is still expected to generate effect. Patients with a mild depression are offered interventions of low intensity (brief interventions such as self-help or counselling). More intensive treatment options are appropriate for patients who do not successfully respond to low-intensity interventions, or for patients who are more severely depressed. Patients with a moderate to severe depression are therefore offered interventions of a higher intensity (psychotherapy and/or antidepressants). Criteria for the different severity levels of depression, mild, moderate or severe, are based on DSM-IV-R criteria [14]. To allocate stepped care adequately, depressed patients have to be identified timely and the severity of the depressive symptoms has to be assessed. Monitoring of the patients’ symptoms is needed for deciding when to step up to a more intensive treatment.

A requirement for the provision of stepped care is the availability of a range of low and high intensity interventions, provided by the GP or in collaboration with other primary or secondary mental health care professionals. Just as in the UK [15], policy in the Netherlands aims at increasing access to low-intensity psychological interventions.

To our knowledge, little research has been conducted to explore the application of depression guidelines in routine primary care. The available studies either offer general information about the quality of care [16-19] or highlight a science-to-practice gap on single indicators, like monitoring of depressive symptoms by GPs [20], recognition and initiation of treatment [21,22] and antidepressant prescribing [23]. Few studies have evaluated the entire stepped care approach, exploring how patients step up to different levels of interventions according to symptom severity, and analysing the factors influencing the organization of this process in daily practice. For example, the implementation of the Increasing Access to Psychological Treatment (IAPT) program was investigated, and the researchers observed significant variation in the levels of implementation in daily practice [15]. Similarly, a stepped care implementation program was examined in the Netherlands, and it was concluded that differing views on depression and depression care within the multidisciplinary healthcare team, lack of resources, and underdeveloped information systems hindered implementation [24]. A qualitative study in UK routine care reported that the quality indicators as imposed by the Quality Outcomes Framework did not match “with the complex reality of general practice” [25]. These studies underline what has been described by Grol and Wensing (2004), namely that implementation of complex treatment approaches, such as stepped care, depends on a complex interplay of factors and overcoming barriers on different levels: the innovation itself, the individual professional, the patient, the organisational context, and the economic and political context [26]. In conclusion, although some information is available on facilitators and barriers to the implementation of guideline based stepped care principles for depression in primary care, limited information is available about the application of these principles in routine care, particularly concerning (relapse) prevention and chronic care.

The aims of this explorative, mostly qualitative study was two-fold. The first aim was to explore the gap between routine care (as provided by GPs and other primary care professionals), and optimal care (as formulated in the depression guidelines). The second aim was to identify facilitators and barriers that affect the provision of optimal care in general practice were identified.

## Methods

### Study design and selection of participants

Considering the exploratory nature of the study aims, a predominantly qualitative approach was adopted to examine thoroughly the depression care as provided by a sample of GPs and the mental health care professionals they collaborated with. This study adheres to the RATS guidelines on qualitative research (http://www.biomedcentral.com/authors/rats). In qualitative research, a purposive sample strategy is recommended when the goal is to generate diversity of opinion and experiences. To this end, six GPs were recruited from three different regions of the Netherlands (north, central and south) where different health insurance companies are active. In a country with mandated universal health insurance coverage, operated by a handful of large commercial health plans, this seemed a relevant factor. In each region, experts in the field of general practice and mental health were contacted and requested to assist in the recruitment of two GPs. They were asked to select one GP working in a practice with preconditions considered relevant for the delivery of stepped depression care and one GP working in a practice without these preconditions. These preconditions were 1. having an MHN or a psychiatric nurse working in the practice to assist the GP in providing mental health care, and 2. having a multidisciplinary team (e.g. comprising a pharmacist, physiotherapist, GP, nurse, primary care psychologist (PCP), and/or a social worker) working on-site, with whom the GP can collaborate in the care for patients. We assumed that the assistance of a MHN or psychiatric nurse, and the proximity of other primary care professionals encourage optimal depression care. The six GPs themselves selected mental health care professionals they collaborated with in depression care to participate in group interviews (see below). This selection procedure led to a total of 22 mental health care professionals that took part in the study.

### Indicators of “optimal care”

To explore the gap between optimal and actual care, we developed a set of indicators representing optimal depression care in five areas: I. Early recognition and indicated prevention; II. Self-management and e-health interventions; III. Diagnosis and treatment according to the stepped care model; IV. Disease management and collaborative care in recurrent or severe or prolonged depression; V. Relapse prevention, rehabilitation and participation. These were the areas that at the time of our study, had just been identified by a national working group for the development of a national care standard for depression, based on available guidelines [27]. The care standard provides a general framework that describes the requirements that good care for depression must meet in terms of both content and process.

In the literature we found indicators that covered most of these five areas of depression care [28-31]. However, for areas III and IV, no or a limited number of indicators were available. In these cases, the researchers themselves (AM, MH) developed four additional indicators. This process resulted in a set of eleven indicators in total, as shown in Table 1. The indicators measure aspects of the health care processes and structures of depression care (see Table 1). An example of a process indicator of area I is ‘Use of a screening instrument in patients who are suspected of having a depression’. This means that the use of a screening instrument was considered to represent optimal care.

**Table 1:** Indicators covering five areas of depression care

**Indicators**	
**I Early recognition and indicated prevention**	
1.1	Use of a screening instrument in patients who are suspected of having a depression
**II Self-management and e-health interventions**	
2.1	Providing self-management and/or e-health interventions to patients with depressive symptoms or a mild depression
**III Diagnosis and treatment according to the stepped care model**	
*IIIa.*	*Diagnosis and symptom severity*
3.1	Measurement of the severity of the depression prior to possible treatment
*IIIb.*	*Applying basic interventions*
*3.2	Providing educational material to patients with depression
*IIIc.*	*Providing stepped care treatment*
3.3	Providing first step and brief interventions to patients with a mild depression^#^
3.4	Providing psychotherapy and/or pharmacotherapy to patients with a moderate to severe major depressive disorder or a recurrent depression^#^
3.5	Systematically monitoring changes in the severity of the depression in patients with a validated instrument
**IV Disease management and collaborative care in recurrent or severe or prolonged depression**	
4.1	Making collaborative care agreements when multiple health care providers are involved in the treatment of a patient with a (severe or prolonged) depression (“who does what”)
*4.2	Making agreements about referral of patients from secondary mental health care to primary care
**V Relapse prevention, rehabilitation and participation**	
5.1	Providing relapse prevention^#^
5.2	Providing ongoing counselling to patients with chronic depression (who are referred back from secondary care)^#^

^#^Indicator that was developed for this study.

*Some indicators belong in more than one area:

- Indicator 3.2 belongs to area II and III.

- Indicator 4.2 belongs to area IV and V.

### Data collection methods

The indicators served as the basis for data collection using a mix of research methods. Routine care was explored with a mix of research methods. Semi-structured interviews were conducted with the six GPs individually (individual interviews), and with the GPs and collaborating mental health care professionals (group interviews). The interviews seemed appropriate to address the experiences and choices that GPs make in routine care, and their perceptions and attitudes on how to improve the quality of depression care. To complement the individual interviews, the GPs completed a self-assessment questionnaire about the actual depression care they provided. Data collection took place from January to May 2012.

#### Individual interviews with the GPs

For the semi-structured individual interviews, an interview guide was prepared, based on the eleven indicators of optimal depression care. This interview guide was pilot tested with an independent GP (an expert in the field of primary mental health care). Final interview items covered routine care provided by GPs to depressed patients, as well as the facilitators and barriers to delivering optimal depression care. Examples were: “How do you recognise depressive symptoms in patients?”, “Do you pay extra attention to the onset of depressive symptoms in specific patient groups?”, “Do you make use of screening instruments?”, and “What is the reason for using/not using them?”.

#### Group interviews with the GPs and mental health care professionals

For the semi-structured group interviews, an interview guide was prepared based on the indicators that focus on allocation of tasks and collaborative care (see Table 1, areas IV and V). The items covered included routine depression care, the roles of the various health care professionals involved in depression care, as well as the facilitators and barriers to collaboration. Examples were: “What common agreements do you have on treatment standards?”, and “Can you easily contact one another to discuss a patient?”.

#### Self-assessment questionnaire for the GPs

The self-assessment questionnaire covered questions on all eleven indicators of optimal depression care. The questionnaire was composed of questions using different rating scales, for example “Are screening instruments used in your practice (yes/no)?”, and “For what percentage of patients that you consider as possibly depressed do you use a validated screening instrument (1-25%; 25-50%; 50-75%; 75-100%)?”. Moreover, the GPs were asked to select indicators that left “the most room for quality improvement in their own practice”. In addition, socio-demographic and professional characteristics of the GPs were collected, such as gender (male/female), age (years), having a special interest in depression (yes/no), having completed training in depression care (yes/no), having participated in a quality improvement project for depression care (yes/no), having a mental health provider available in the practice (yes (disciplines)/no), and having documented collaboration agreements with specialty mental healthcare organizations (yes/no).

### Data collection

All interviews took place at the GPs’ office. Each individual interview lasted around 60 minutes and was conducted by one of the researchers (AM). The self-assessment questionnaire was handed out to the GPs after each individual interview. The group interviews took around 90 minutes and were conducted by two researchers in varying pairs (AM, MH, PvS, and JN).

### Data analysis

The semi-structured interviews were audiotaped and notes were taken. To order the data, thematic coding was used with the support of MAXQDA [32], a software programme for qualitative analysis. A coding tree was built around the indicators (Table 1), and text fragments were coded by one of the researchers (AM). In between interviews content analysis was performed by at least two researchers (AM and GF), generating new insights and questions for subsequent interviews. Text fragments with the same code were compared on agreement and differences. The answers to the self-assessment questionnaires were presented in a table for each GP individually (numbers and percentages).

## Results

### Study population

Table 2 shows that the purposive selection of the six GPs had been successful. Three GPS had practice conditions considered relevant for the delivery of stepped depression care (i.e. the assistance of a MHN or psychiatric nurse, and the proximity of other primary care professionals), while the other three GPs had not. The GPs that had organisational conditions relevant for stepped depression care more often received training on depression, participated in a quality improvement project, and had collaboration agreements with mental healthcare providers in primary care (Table 2). Most of the 22 mental health care providers who were selected by the GPs for the group interviews were working in primary care (mostly MHNs and PCPs); only two were from secondary mental health care (psychiatrists).

**Table 2:** Characteristics of the GPs and their practices

**Characteristics per GP**	**GP 1**	**GP 2**	**GP 3**	**GP 4**	**GP 5**	**GP 6**
Organisational preconditions for optimal depression care available*	No	No	No	Yes	Yes	Yes
**Region of the Netherlands**	North	Middle	South	North	Middle	South
**Sex**	Woman	Man	Man	Woman	Woman	Man
**Age**	47	39	56	60	43	58
**Specific interest in depression**	No	Yes	No	Yes	Yes/No	No
**Training on depression (last 3 years)**	No	Yes	No	Yes	Yes	Yes
**Participation in quality improvement project on depression**	No	No	No	Yes	Yes	Yes
**Mental health care providers on-site**	MHN	No	No	MHN, PCP, PP	MHN, PCP, PP	MHN (2x)
**Document on collaboration within primary care**	No	No	No	Yes (limited)	No	Yes (limited)
**Document on collaboration with secondary care**	No	No	No	No	No	No
**Other disciplines in group interview**	MHN, PCP, psychiatrist	SPN, PCP (2x), GSW, AOP	Psychiatrist, pharmacist, GSW, physio-therapist	MHN, PCP (2x), PP	MHN, PCP	MHN (2x), PCP, GSW

*Abbreviations*: *AOP* Advisor for Older People, *GSW* General Social Worker, *GP* General Practitioner, *MHN* Mental Health Nurse, *PCP* Primary Care Psychologist, *PP* Psychosomatic Physiotherapist, *SPN* Social Psychiatric Nurse.

*These preconditions were 1. having an MHN or a psychiatric nurse assisting the GP, and 2. having a multidisciplinary team working on-site, with whom the GP can collaborate in the mental health care for patients.

### Research question 1: Is there a gap between routine care and optimal care?

The overall findings are reported for the five areas of depression care successively. For each area, first optimal care is briefly described (based on five areas of depression care contained in the care standard for depression) [27], and subsequently routine care is described (based on the questionnaire and the results of the interviews, see Table 3).

I. Early recognition and indicated prevention.

Optimal care. Care is considered optimal when GPs are attentive to signals of depression in their patients. Screening instruments may be used when depression is suspected. Prevention may consist of self-help or self-management to those with depressive symptoms (which is called indicated prevention).

Routine care. Our results showed that the GPs were attentive to signals of depression in actual daily practice, for example in patients with vital features of depression or in patients who present numerous physical symptoms. Some GPs and MHNs used a screening instrument (see also Table 3), mostly the Four-Dimensional Symptom Questionnaire (4DSQ) [33] and found it a useful tool to discuss the symptoms with patients and to distinguish the depression symptoms from distress, somatisation or anxiety symptoms. However, most GPs mainly relied on their clinical judgment in recognising depressive symptoms. Indicated prevention consisted merely of watchful waiting, and sometimes the GPs asked patients to return for an additional consultation.

II. Self-management and e-health interventions.

Optimal care. Care is considered optimal when self-management interventions (e.g. self-help groups, running therapy, bibliotherapy) and e-health interventions are available for patients that seek help for depressive symptoms or a mild depression.

Routine care. The results indicate that in actual daily practice the GPs supported self-management mainly by giving general lifestyle advice (e.g. on healthy diet and exercise). Patients were seldom referred to group courses or to running therapy. E-health interventions were provided sparsely by both the GPs and the other participating mental health care providers (see also Table 3). This was mainly due to unfamiliarity with reliable e-health instruments and websites, and to uncertainties about the appropriateness of e-health interventions for depressed patients and about how to ensure a working relationship with a care provider during an e-health intervention.

III. Diagnosis and treatment according to the stepped care model.

Optimal care. Care is considered optimal when the diagnostic process includes a clinical assessment. To determine the treatment policy it is important to differentiate in depression severity and duration. An assessment tool can be used to assess the severity of the symptoms. When a diagnosis of depression is established, basic interventions should be offered to all patients (e.g. psycho-education and lifestyle advice). Patients with a mild depression should be offered brief interventions, such as counselling, psycho-social interventions and Problem Solving Treatment (PST), in which the patient’s competence and coping skills are strengthened. Psychotherapy or pharmacotherapy should be available for patients with a moderate to severe or recurrent depression. To evaluate the treatment effect, a patient’s symptoms of depression should be monitored regularly, preferably with the help of an instrument.

Routine care. Most of the time, the GPs that participated in our study found it difficult to diagnose a depression as such. This was often due to the existence of various other problems and symptoms patients experienced in addition to the depressive symptoms. When making a depression diagnosis, the GPs placed a patient into one of three subgroups: those who have depressive symptoms, those with a depression and those with a complex depressive disorder. Some GPs acknowledged that they found it difficult to distinguish exactly between depressive symptoms and a depression; they perceived it as a continuum. The GPs and other participating primary care professionals provided basic interventions to patients with a depression, such as psycho-education and individual lifestyle advice (e.g. engaging in external and social activities).

GP2: I advise patients to exercise regularly, as this often reduces psychological symptoms. [..] And I give them a daily schedule, as people need to develop enough activities. I refer to this schedule regularly with the patient, and they tell me what went well and what didn’t go so well.

The GPs applied the general idea of stepped care. Medication was not the first step in the treatment of patients with a mild depression. Besides the severity of symptoms, patients’ preferences, their former experiences with interventions, and their personal, financial and social circumstances played an important role in the clinical decision making of the GPs. The indication criteria for various depression treatments differed between the participating GPs and were seldom applied in a structured manner. In general, most GPs started with counselling or psychosocial interventions (see also Table 3). Since the GPs are not only central providers but also act as brokers, the GPs sometimes made a referral to the MHN or PCP, who provided these interventions in a more structured way. PST, however, was rarely provided. When the treatment effect was not satisfactory the GPs stepped up to more intensive interventions. They offered (or referred to) pharmacotherapy or psychotherapy (see also Table 3) - interventions with which the GPs are familiar. Most GPs provided pharmacotherapy themselves. The GPs did not systematically use instruments to assess the initial severity of the depression or to monitor changes in the severity of the depressive symptoms during treatment (see also Table 3). Reasons for this were doubts the GPs had about the value of the instruments, unfamiliarity in using them and the necessary time investment. Most of the MHNs were more familiar with the use of instruments and had positive experiences in using them for assessing and monitoring depressive symptoms. The GPs had the impression that the group of patients presenting with a complex and severe depression was very small. Severely depressed patients with, for example, personality disorders, psychotic features or with suicidal behaviour were directly referred to secondary mental health care services.

IV. Disease management and collaborative care in recurrent or severe or prolonged depression.

Optimal care. Care is considered optimal when collaborative care agreements are made regarding allocation of tasks where multiple providers are involved in the treatment of a patient with a (severe or prolonged) depression. Agreements should also be made when patients are referred back from secondary to primary care.

Routine care. The results demonstrated that none of the GPs in our study had collaborative care agreements with secondary mental health professionals. Few patients were referred directly to secondary mental health care services; only those with a severe and complex depression, or those who had previously received treatment in secondary care were referred. Rarely were patients referred back from secondary care to the GP for aftercare (see also Table 3). Considerable between-practice variation was found in the collaboration within primary care; some GPs closely collaborated with MHNs or with PCPs, while other GPs only made referrals but did not collaborate, or rarely consulted other primary mental health providers (see also Table 3).

V. Relapse prevention, rehabilitation and participation

Optimal care. Care is considered optimal when the aim of treatment shifts to preventing relapse after remission from a depressive episode. The patient and care provider make a relapse prevention plan, and the care provider can offer medication and/or specific psychological interventions, such as preventive cognitive group therapy, maintenance interpersonal therapy and mindfulness based cognitive therapy. In chronically depressed patients, combination therapy is preferred (i.e. a combination of pharmacotherapy and psycho-therapy), supplemented with rehabilitation interventions.

Routine care. Our results showed that relapse prevention and care for patients with chronic depression mostly comprised continuation of antidepressant medication with low frequency contacts (see also Table 3). The primary care providers were not familiar with specific psychological interventions for these patients.

GP6: I don’t do much with patients who have a chronic depression. There are patients who merely receive their medication. Sometimes I see them for another problem. Sometimes I address it by asking how they are doing. But that’s all.

**Table 3:** Results of the self-assessment questionnaire for GPs on eleven indicators of optimal care

	**GP 1**	**GP 2**	**GP 3**	**GP 4**	**GP5**	**GP 6**
**Organisational preconditions for optimal depression care available**^$^	No	No	No	Yes	Yes	Yes
**1.1 Use of a screening instrument in patients who are suspected of having a depression**	Yes*	Yes	No*	Yes	No	Yes
	25-50%^#^	1-25%	0%	75-100%	0%	50-75%
**2.1 Providing self-management and/or e-health interventions to patients with depressive complaints or a mild depression.**	Yes	Yes	No	Yes	No	Yes
	1-25%	75-100%	0%	25-50%	0%	25-50%
**3.1 Measurement of the severity of the depression prior to possible treatment**	Yes	No	No	No	No	Yes
	1-25%	0%	0%	0%	0%	50-75%
**3.2 Providing educational material to patients with depression**	Yes	Yes	No	Yes	Yes	Yes
	75-100%	1-25%	0%	1-25%	1-25%	25-50%
**3.3 Providing first step and brief interventions to patients with a mild depression**	Yes	Yes	Yes	Yes	Yes	Yes
	50-75%	75-100%	25-50%	75-100%	25-50%	50-75%
**3.4 Providing psychotherapy and/or pharmacotherapy to patients with a moderate to severe major depressive disorder or a recurrent depression**	Yes	Yes	Yes	Yes	Yes	Yes
	50-75%	75-100%	75-100%	50-75%	25-50%	75-100%
**3.5 Systematically monitoring changes in the severity of the depression with a validated instrument**	No	No	No	No	No	yes
	0%	0%	0%	0%	0%	25-50%
**4.1 Making collaborative care agreements when multiple health care providers are involved in the treatment of a patient with a (severe or prolonged) depression (“who does what”)**	Yes	No	No	Yes	Yes	No
	25-50%	0%	0%	?	25-50%	0%
**4.2 Making agreements about referral of patients from secondary mental health care to primary care**	No	No	No	No	No	Yes
**5.1 Providing relapse prevention**	Yes	Yes	Yes	Yes	Yes	Yes
	25-50%	75-100%	75-100%	?	25-50%	1-25%
**5.2 Providing ongoing counselling to patients with chronic depression (who are referred back from secondary care)**	No	No	No	Yes	No	No
	0%	0%	0%	50-75%	0%	0%

*yes/no = GPs indicated whether or not they delivered specific care.

^#^% = which proportion of patients within the target population received that specific care (? = does not know).

^$^These preconditions were 1. having an MHN or a psychiatric nurse assisting the GP, and 2. having a multidisciplinary team working on-site, with whom the GP can collaborate in the mental health care for patients.

In general, the MHNs and PCPs offered more structured and planned follow-up contacts compared to the GPs.

### Research question 2: What factors influenced the delivery of optimal care?

The GPs and the other participating mental health care providers indicated many factors influencing the delivery of optimal care for depression. Table 4 gives an overview of the barriers and facilitators that were put forward by the study participants, categorised according to the five levels distinguished by Grol and Wensing (2004) [26]: the innovation itself, the professional, the patient, the organisation and the economic and political context. Table 4 also shows for which elements of depression care the participants experienced barriers or facilitating factors.

**Table 4:** Summary of facilitators and barriers to optimal care for depression

**Factors on the level of the …**	**Facilitators**	**Barriers**
Innovation itself	• A screening or monitoring instrument can help in talking with patients about their symptoms (indicator 1.1)	• Unclear for which patient subgroups certain interventions are appropriate (indicator 2.1)
Individual professional	• Having a special interest in mental health problems (indicator 3.3)	• Contentment with the current routine care (the GPs considered the provision of pharmacological and psychological interventions the most important elements of depression care, and they could provide these interventions to their patients) (indicators 3.3, 3.5, 5.1)
	• The perceived proximity of primary mental health care providers (indicators 4.1 and 4.2)	
	• The availability of instruments or interventions that have practical clinical usefulness (indicators 1.1, 2.1, 3.1, 3.3, 3.5)	
		• Unfamiliarity with certain interventions or tools (e.g. e-health interventions, relapse prevention, interventions for patients with chronic depression) (indicators 1.1, 2.1, 3.1, 3.2, 3.3, 3.5, 5.1, 5.2)
Patient	• Patient preferences for certain interventions (indicator 2.1, 3.3, 3.4)	• Not having internet access, e-health interventions therefore unavailable (indicators 2.1, 3.2)
	• The GP cannot lose sight of the patients; they go to the GP now and then anyway for other reasons than psychological problems (indicators 5.1, 5.2)	
		• Costs associated with health care use (patients prefer care that is without charges) (indicator 3.3)
		• Poor adherence to treatment (indicator 5.2)
Organisational context	• An MHN is available in primary care (who has, for example, more time to assess and monitor symptom severity systematically) (indicators 1.1, 3.1, 3.5, 4.1, 5.1, 5.2)	• Lack of collaboration between primary care and secondary mental health care (e.g. no agreements in place with secondary care about care delivery) (indicators 3.4, 4.1, 4.2, 5.1)
	• Easy access to interventions (indicator 3.2)	
	• Close collaboration and regular consultation within primary care (indicators 1.1, 2.1, 3.3, 5.1)	• Lack of proper and timely reports from secondary to primary care about referred patients (indicators 4.1, 4.2)
	• Participation in a quality improvement project on depression care (indicators 1.1, 3.1, 3.3, 3.5, 4.1, 5.2)	• Investing in education, time and effort to achieve knowledge and experience (indicator 5.2)
	• Having agreements on indication criteria and treatment policy within primary care (indicators 1.1, 3.1, 3.3, 4.1)	
Economic and political context	• Financial incentives to improve collaboration between primary and secondary mental health care (indicators 4.1, 4.2, 5.1, 5.2)	• The different financial structures for primary and secondary care (indicators 2.1, 4.1, 4.2, 5.1)
		• Financial contributions that patients have to pay for certain care providers (indicators 3.3, 3.4, 4.1, 4.2)
	• Financial incentives to promote the referral of patients back to primary care when appropriate (indicator 5.1)	
		• Lack of incentives from the professional association of GPs (indicators 3.2, 5.2)

Overview of the barriers and facilitators that were put forward by the participants, categorised according to five levels [26]: the innovation itself, the individual professional, the patient, the organisation and the economic and political context. A reference to one or more indicators of the indicator set (Table 1) is given, to illustrate for which element of care the influencing factor was put forward by the study participants.

A facilitator *on the level of the innovation itself* was the practical clinical usefulness of an instrument, e.g. the screening instrument 4DSQ helped the MHN to talk with patients about the depressive symptoms they were experiencing. Barriers *on the level of the individual professional* were being content with current routine depression care (and thus not being convinced of the necessity to improve care), and being unfamiliar with certain interventions (e.g. e-health interventions or relapse prevention) or tools (e.g. instruments to assess and monitor depressive symptoms). Having a special interest in mental health problems was considered a facilitator. Barriers *on the level of the patient* were poor adherence to treatment (e.g. antidepressant therapy), and not having internet access, which is a necessity for e-health interventions. A facilitator *on the level of the economic context* was the availability of incentives from health insurance companies (e.g. for participation in quality improvement projects on depression). Charges for patients who received depression care from PCPs or secondary mental health care, and lack of reimbursement for GPs to hire a MHN were put forward as barriers. Different financial structures and the lack of compensation for time spent on communication and collaboration between primary and secondary mental health care were factors that hindered stepped mental health care. Finally, a facilitators *on the level of the organisational context* was close collaboration and regular consultation within primary care. In our study, GPs were enrolled that showed diversity on this criterion. The results of this study indicate that GPs with practice conditions that were considered relevant for the delivery of stepped depression care delivered more elements of stepped care; the available MHNs made use of screening instruments and MHNs as well as PCPs assessed and monitored depressive symptoms. Thus, close collaboration with MHNs or PCPs seemed favourable for delivering optimal care. Another facilitator *on the level of the organisational context* was having good collaborative care agreements between GPs and MHN and PCP, and receiving proper and timely patient reports from secondary care about referred patients.

#### The improvement potential in areas I-V according to the GPs

In the self-assessment questionnaire, and also in the interviews, most GPs indicated that the quality of care for depressed patients could be improved mostly by the strengthening of primary mental health care. Although they also indicated that collaboration with secondary care left the most room for improvement, the GPs emphasised the importance of close collaboration with other primary mental health care providers. Furthermore, GPs indicated that the provision of e-health interventions, interventions for relapse prevention, and the care for patients with chronic depression could be improved.

All participating GPs were dissatisfied with the information sharing by secondary mental health care regarding referred patients, except for one GP:

GP6: The discharge letter contains advice about the treatment, but also advice that was given to the patient. So I know what to do if things should go wrong. If and how I should continue medication, whether I should follow up on the patient - it’s all in the letter.

Collaboration with secondary mental health care concerning patients with complex and chronic depression was unsatisfactory.

GP5: When I send a patient with appendicitis to the hospital, the patient is referred back to me for removing the stitches, for example. So then you have joint responsibility as clinicians. But if I refer a patient to secondary mental health care, there is no such thing as joint responsibility. The secondary mental health care is like an island where we do not belong.

## Discussion

### Main findings

On the one hand, the results can be interpreted to mean that the GPs and their primary care colleagues embraced the concept of a general stepped care approach in the care for depressed patients. That is, the GPs indicated to differentiate between three severity levels of depression (depressive symptoms, depression, and severe/complex depression), and to provided low-intensity interventions when possible (e.g. counselling), and high-intensity interventions only when necessary (e.g. psychotherapy and pharmacotherapy). Patients with a severe and complex depression were referred directly to secondary care.

On the other hand, the findings indicated that several essential elements of the stepped care approach were not daily practice. Several low-intensity psychological interventions such as PST, self-management and e-health interventions were rarely provided to patients with depressive symptoms or mild depression. Collaboration with secondary mental healthcare was virtually non-existent, and specific psychological interventions for relapse prevention or for chronically depressed patients were not available. Last but not least, assessing the severity of depressive symptoms in patients with a diagnosed depression and monitoring the course of the symptoms are important requirements of the stepped care approach, but were not systematically applied by the GPs.

There is a wide range of facilitating and impeding factors for the provision of optimal depression care. These factors were related to specific tools or interventions, individual professionals, patients, the organisation and the financing of (mental) health care. Close collaboration with other (mental) health care professionals involved in the care for depressed patients was considered an important factor for improvement by nearly all GPs.

### Strengths and limitations

A strength of our study is that we investigated the whole continuum of depression care, from prevention of depression to chronic depression care. It also included some innovative interventions, such as e-health and self-help. Most other research focused on patients that are already identified, and chronic care is not included. Another strength of the study was that methodological triangulation was employed as a research method. It is likely that we got a clear picture of the routine depression care, as provided by the participating GPs, by combining the qualitative results of the individual and group interviews with the quantitative results of the self-report questionnaire.

A limitation of our study was its rather small study sample of six GP practice teams. However, we did not try to enroll GPs that were representative of GPs in the Netherlands, but that offered the existing spectrum of more or less optimal depression care. We aimed for maximum diversity, not representativeness. Therefore, the GPs were chosen purposively and not at random. In qualitative research this is considered an appropriate sampling strategy to provide diversity of opinions and experiences.

Another study limitation is the lack of quantitative data on care consumption in the selected practices, based on registrations in the electronic medical record (EMR) or on claims data. However, this was not an option since this level of detailed information on depression care is not available in existing EMRs or any other registry in the Netherlands.

### Relation to existing literature

Some findings of this study have, to our knowledge, never been reported before. We believe this is the case for the information about how GPs manage relapse prevention for depression and what kind of care they deliver to patients with chronic depression in daily practice. There is ample research that relates to other findings of our study.

With regard to the use of instruments, it was reported in a Canadian study that GPs generally used clinical intuition with few clinical tools to detect, diagnose and monitor mental disorders [34]. A recent Dutch study also found that GPs do not often use a screening instrument for depression or an instrument to assess the severity of a newly diagnosed depression [35].

In our study GPs indicated that patients’ preferences, former experiences with mental health care, and financial capacity play an important role in allocating treatment. Perceived patients’ preferences may impede stepped care allocation while severity assessment is positively associated with allocating stepped care [35]. Another Dutch study showed that patients’ preferences as well as the education level of the patient are more strongly associated with the delivery of guideline-concordant care than clinical need factors [36]. Investigators in the UK found that GPs prescribe medication based on their clinical judgment of the severity of the depression [21]. They also stated that GPs’ judgment of severity is not always consistent with a validated depression scale and they therefore advocate better ways to appropriately allocate treatment.

Comparable to the results of our study, other Dutch studies found that the provision of certain structured low-intensity interventions, like e-health or self-help interventions for depressive symptoms and mild depression in general practice, is limited [24,35]. Likewise, a Canadian study reported that GPs most frequently offered pharmacotherapy, support therapy, and psycho-education to patients with mental health disorders [34].

Strengthening mental health care in primary care is a subject that receives growing attention from researchers. GPs in our study expressed a need for close collaboration with a limited number of mental health care providers, as was found in another Dutch study [37]. Structural collaboration between GPs and mental health care professionals in primary and secondary care improved the assessment of depression severity, which was in turn associated with allocating stepped care for depression [35]. A Canadian study also found that increased collaboration in mental health care between primary care providers fostered better mental health treatment [34]. Another study reported that GPs in the UK had difficulty referring to mental health care as it was unclear what treatments were offered by different mental health care providers [25].

Poor collaboration between primary and secondary care appears to be a rather consistent finding across countries like the UK, Australia and Canada [25,38,39]. A Canadian study found numerous factors hindering collaboration, which correspond to our findings: lack of resources, lack of training, time and incentives for collaboration, and inappropriate payment modes [39]. The Canadian researchers state that a culture of collaboration has to be encouraged as comprehensive services and continuity of care are key recovery factors of patients with mental disorders [39].

### Recommendations

Strengthening primary care is high on the agenda of many western countries. Since the burden of disease caused by depression is expected to rise globally, the role of primary care clinicians in dealing with this conditions will be even more pivotal than it is today. The results of this study suggest that primary care clinicians in the Netherlands, when asked about their depression work, indicate to largely follow evidence-based recommendations. Still, when detailed quality indicators are used to assess their work, there seems to be room for improvement in the way clinicians screen and monitor patients, treat mild cases of depression, give attention to relapse prevention and chronicity, and collaborate with colleagues within and across settings. These improvements might not occur spontaneously which leads to the main message of our study that, in order to create highly effective and efficient primary mental health care, stakeholders should invest and support clinicians to improve their care. This can be done by increasing knowledge and skills, by paying for staff to assist GPs, by introducing financial incentives that rewards collaboration so that less unneeded referrals from primary care to secondary care occur.

The findings of this explorative qualitative study should not be considered as hard (statistical) evidence of the quality of depression care by GPs in the Netherlands. Therefore, future quantitative and qualitative research could confirm and build on the findings of this explorative study. Preferably these studies should include the perspective of patients and specialised mental health clinicians since they are all partners in depression care.

## Conclusion

The management of depression in Dutch primary care, as reported by clinicians, seems in line with stepped care principles, although it can be further improved by applying more elements. These concern in particular the use of assessment and monitoring instruments, low-intensity psychological interventions such as (guided) self-help, e-health interventions and PST, and improving the collaboration between providers in primary and secondary care. Clinicians and policy makers could take these into account in the design and financing of improvement programs.

## Abbreviations

GP: General practitioner; MHN: Mental health nurse; PCP: Primary care psychologist; PST: Problem solving therapy.

## Competing interests

The authors declare that they have no competing interests.

## Authors’ contributions

MH drafted the manuscript. JN and GF conceived the study. MH, GF, PvS, and JN designed the methods. MH, AM, PvS, and JN collected the data. AM, GF and MH performed the qualitative content analysis. AM, GF, PvS, and JN helped to draft the manuscript. All authors have read and approved the final manuscript.

## Pre-publication history

The pre-publication history for this paper can be accessed here:

http://www.biomedcentral.com/1471-2296/15/5/prepub
